# De Senectute

**Published:** 1905-09

**Authors:** 


### "De Senectute."

IN THE August, 1905, issue of this JOURNAL we published the address of Dr. Charles G. Stockton, of Buffalo, delivered at Portland, Ore., before the American Medical Association during its fifty-sixth annual meeting, the subject being "The Delay of Old Age and the Alleviation of Senility." In the same issue we made editorial comment thereon, though conscious at the time of doing the orator and his oration very inadequate justice.

However, the *Medical Standard*, Chicago, has come to our aid and in its August edition under the title "De Senectute"—most appropriately chosen—prints an analysis of Dr. Stockton's oration that is unexcelled for beauty of diction or comprehension of the subject. We take great pleasure in presenting it to our readers in full.

### DE SENECTUTE.'

If the recent convention of the American Medical Association were productive of no other result than the calling forth of Dr. Stockton's masterly oration on old age, it would not have convened in vain. In our opinion this production easily ranks first among the array of oratory which graced the gathering at Portland. Indeed, we do not believe we are overrating it when we say that it is worthy to take its place with the classic utterance on the same subject by Cicero, and unless we are much mistaken

Dr. Stockton has, as a result of his effort, by this time awakened to find himself famous. It is rarely that a medical man rises to such a lofty and comprehensive viewpoint of his subject; still more rarely that he unites such loftiness of survey with such breadth of treatment; such wealth of imagination with such moderation and sanity; such beauty with such practicality; such philosophy with such science.

Perhaps the masterly qualities of Dr. Stockton's address stand out the more conspicuously in contrast with the childish small-talk on the same topic, to which we have recently been treated in quarters where we might reasonably have expected more dignified utterance or discreet silence. We have been regaled of late with all manner of panaceas,—ranging all the way from goat-lymph to microbe cultures,—for the prevention of old age. But while Nero fiddled Rome was burning, and while these gentlemen have been shouting their cure-alls from the market place senility has been steadily creeping beyond its legitimate bounds, and becoming more and more premature, until real old age, the old age of which Dr. Stockton speaks, the mellow evening of a ripe and rounded life, is rapidly becoming a thing of the past.

It is this vanishing splendor of a mature and finished life that Dr. Stockton would recall and reinstate. He has no wild scheme to offer for the evasion or abortion of old age, for he wisely recognises that a normal old age is a thing to be courted, not to be evaded. He pleads only for a physiological senility, instead of a pathological one; for a mature, in place of a premature old age. On the other hand, he indulges in no hopeless beating of the air, but lays a calm and intelligent finger on the root of the trouble, and points a practical and rational way of remedy.

Altogether it is one of the loftiest and most stirring appeals ever made to modern science. Rightly understood it compasses the whole duty of the physician, to which all other branches of his calling are tributary. It is a call, not only to the preservation of physical existence, but to the moulding of mental and moral perfection; not merely to the lengthening of the span of human life (which is in itself an ignoble consummation), but to the restoration of its crowning glory. If it be true that he who makes two blades of grass to grow where but one grew before is a benefactor to his race, he who makes a flower to spring in the place of a weed is more than a benefactor—he is an uplifter and a transformer. To such a high office does Dr. Stockton invite us.

---

Dr. T. D. Crothers, of Hartford, Conn., superintendent of Walnut Lodge Hospital, has accepted an invitation to deliver the first oration in the Norman Kerr memorial lectureship, at London, Eng., October 10, 1905. Dr. Kerr will be remembered as an eminent London physician who made a special study of inebriety, alcoholism and other drug disorders. He wrote several excellent books on this subject, and was instrumental in securing the enactment of laws for the control of inebriates and the promotion of hospitals for their care throughout Great Britain. He founded the British society for the study of inebriety, in 1884, and this society and his friends have organised a memorial lectureship for yearly orations on his life and work. It is a very pleasant recognition of the progress of medical science in this country, that an American physician should be invited to deliver the first lecture.

---

MR. JOHN W. BANNING, who poses as president of the "Atlantic School of Osteopathy," was recently arrested on a charge of violating the university law of the state of New York. The "school" operates under a business charter obtained from New Jersey. It advertised commencement exercises to be held in the latter part of June and issued so-called diplomas to about twenty persons, without authority from the education department of the state. Banning was released on bail and the case is set down for a hearing August 31, 1905. The board of censors of the Medical Society of the County of Erie is the complainant and Mr. Assistant District Attorney Abbott is conducting the case for the people.

---

DR. STOCKTON, in his oration at Portland, referred to the benefits dentists had conferred on mankind, and pointed out how the nutrition of the aged had been improved by good teeth, for which, in large measure the dentists were to be credited. But even of greater importance is a good dentifrice, because everybody can use a tooth powder, whereas but few comparatively can employ a dentist.

Calox is the dentifrice, par excellence, for rich and poor alike. It is cleanly, it is antiseptic, it prevents decay by destroying germs, it renders the brush aseptic, and keeps the mouth wholesome. It is not a secret preparation but a scientific product of the chemical laboratory, and it is within the reach of the slender purse.

---

THE Grosvenor Public Library has improved its accommodations for physicians by removing its medical section to the basement of its building at Franklin and Edward streets. Commodious rooms have been newly furnished, which are light and well ventilated. Physicians are invited to inspect the new medical quarters and to avail themselves of the pr vileges of the library.

The annual excursion of the Medical Society of the County of Erie was given Tuesday, August 15, 1905. The day was unpropitious and the attendance was comparatively small.

